# Surgical outcomes in children under 10 years old in the treatment of congenital scoliosis due to single nonincarcerated thoracolumbar hemivertebra: according to the age at surgery

**DOI:** 10.1186/s13018-021-02865-w

**Published:** 2021-12-20

**Authors:** Haixia Li, Zhiming Chen, Bo Gao, Jiaxu Wang, Shuilin Shao, Jigong Wu

**Affiliations:** Department of Spine Surgery, Chinese People’s Liberation Army Strategic Support Force Characteristic Medical Center, Beijing, 100101 China

**Keywords:** Congenital scoliosis, Thoracolumbar hemivertebra resection, Posterior approach, Pedicle screw fixation, Distal adding-on

## Abstract

**Purpose:**

Hemivertebra is one of the common pathogenesis of congenital scoliosis. The timing of operation is undefined. Our study compared the surgical outcomes in children under age 10 years with scoliosis due to single nonincarcerated thoracolumbar hemivertebra according to the age at surgery.

**Methods:**

From January 2009 to August 2017, we retrospectively investigated 34 consecutive cases of congenital scoliosis treated by posterior hemivertebra resection and fusion with pedicle screw fixation. All cases were divided into two groups according to the age at surgery and followed-up for at least 2 years, group 1 (≤ 5 years old), and group 2 (5 to 10 years old).

**Results:**

The mean Cobb angle of the main curve was improved from 48.58° to 15.53° (68.05%) in group 1, and from 43.73° to 11.33° (75.43%) in group 2. The segmental curve was improved from 44.16° to 11.53° (74.64%) in group 1, and the segmental curve was consistent with the main curve in group 2. The mean segmental kyphosis was improved from 27.50° to 8.42° (67.40%) in group 1, and from 29.00° to 5.00° (84.73%) in group 2. Five patients developed distal adding-on, and four patients were found proximal junctional kyphosis during the follow-up.

**Conclusion:**

Not all the deformities caused by single nonincarcerated thoracolumbar hemivertebra would progress greatly with the spinal growth. No significant statistical differences were found in the coronal and sagittal correction rate between the two groups. A limited delayed surgery after 5 years but before 10 years of age with close follow-up can achieve satisfied results.

## Introduction

Hemivertebra is one of the common pathogenesis of congenital scoliosis especially in early onset scoliosis, and it is difficult to predict the prognosis. Some of them tend to create a wedge-shaped deformity during spinal growing period. McMaster and David found that the curve progression and the ultimate severity of the curve produced by a hemivertebra (HV) depends on the rate of deterioration and the severity of the spine deformities caused by HV depended mainly on the type, site, number, and relationship of HV to each other [1]. Single nonincarcerated HV will most likely to result in significant deformity that can progress during growth [2, 3], besides, hemivertebra located in the lower thoracic and thoracolumbar spine are more prone to cause deterioration of the spine’s curvature and therefore earlier surgical intervention is suggested in most cases [4].

Early surgery in young children prevents the development of severe local deformities and secondary structural curves, thus allowing for normal growth in the unaffected parts of the spine [5], while the timing of operation is undefined, there are reports in the literature that achieved good result in children under age 10 years with congenital scoliosis [6–8], also in those before 5 years old [9, 10]. There are no reports in the literature regarding surgical outcomes by the age at the time of surgery in children under age 10 years with congenital scoliosis caused by a certain type of hemivertebra.

The purpose of this study is to compare the surgical outcomes in children under age 10 years with scoliosis due to single nonincarcerated thoracolumbar hemivertebra undergoing posterior hemivertebra resection and fusion with pedicle screw fixation.

## Materials and methods

Inclusion criteria of this study consisted of: (1) age < 10 years old at the surgery; (2) congenital deformity due to single nonincarcerated thoracolumbar hemivertebra; (3) surgery performed by posterior hemivertebra resection with transpedicular instrumentation; (4) at least 2 years’ follow-up with complete radiographic and clinic data. Patients with age more than 10 years old, previous spinal surgery, complex congenital spinal deformity, or syndromic scoliosis were excluded.

A total of 34 consecutive patients treated in our hospital from January 2009 to August 2017 were recruited in this study. All surgeries were performed by two of the authors. The institutional review board of our hospital approved this study before data collection. The patients included 10 females and 24 males, aged from 26 to 119 months old. Of the 34 patients, 4 cases had congenital cardiac malformation, 1 case accompanied with tethered cord syndrome. All the cases were divided into 2 groups according to age for the analysis: group 1 (≤ 5 years old), and group 2 (5 to 10 years old).

Before surgery, a complete physical examination and radiographic evaluation were performed, including standing long cassette anterior–posterior (AP) and lateral (L) X-ray, CT scans with reconstruction to evaluate the shape and position of the hemivertebra and the anatomy around the pedicles, the locations of hemivertebra were all in the thoralumbar (T10-L2); MRI to determine the presence of spinal cord anomalies. All curve parameters were measured using the Cobb method described by Ruf and Harms [11]. The segmental main curve, total main curve, and compensatory curve were measured in the coronal plane. The segmental kyphosis (SK), thoracic kyphosis (TK), and lumbar lordosis (LL) were measured in the sagittal plane in standing AP and L X-ray of the whole spine preoperatively, postoperatively, and at the latest follow-up. Clinical information were reviewed to obtain the age, sex, BMI, operative time, intraoperative blood loss, fusion levels, follow-up time and complications during peri-operative and follow-up periods. The percentage of intraoperative blood loss to estimated blood volume (EBV) was used for the evaluation of blood loss, and the EBV was calculated by body weight (kg) *75 ml/kg [12, 13].

### Operative technique

The surgical technique was performed as earlier descriptions [11, 14]. Transpedicular instrumentation and hemivertebra resection was performed by a posterior-only approach with fusion of the adjacent vertebrae (usually one level above, and one below). More pedicle screws and long fusion were needed in some patients with structural compensatory curves, pronounced kyphoscoliosis, or when the bisegmental instrumentation was not strong enough to close the gap after hemivertebra excision. We put a temporary rod attached to the concave pedicle screws which can provides stability to the spine and possibly avoids microtrauma to the cord while the decancellation procedure was carried out. Subsequently, hemivertebra and the discs adjacent to the hemivertebra were removed, and the vertebral end plates were debrided down to bleeding bone. Thereafter, the rods were placed and compression forces were applied gradually on the convex side and sometimes on the both sides until the gap left after the resection closed completely. If a void remained, cancellous bone was used to fill the gap, and a titanium mesh cage was used in one case to fill the large osteotomy gap and correct the segmental kyphosis after hemivertebra resection. Somatosensory evoked potential (SEP) and motor-evoked potential (MEP) were used intraoperatively.

### Statistical methods

Statistical analyses were performed using SPSS statistics software 20.0 (IBM, NY). Paired t tests were used to assess the differences of radiographic parameters between pre- and postoperative and postoperative and the last follow-up, the independent samples t test was performed to analyze the differences between two groups. A *P* value of less than 0.05 was considered statistically significant.

## Results

General data of the patients are shown in Table 1. There were 34 patients included in our study. All the patients were divided into two groups according to the age at surgery as earlier descriptions. The mean operating time was 163.42 min (75–242 min) in the group 1, and 167.73 min (120–245 min) in the group 2. The mean blood loss during the surgery was 260 mL (50–600 mL) in the group 1, and 496.67 mL (100–1600 mL) in the group 2, the difference between the two groups was statistically significant (*P* = 0.025), and the difference of the mean fusion levels of the two groups was also significant, 2.68 (2–4) in group 1 and 3.53 (2–5) in group 2 (*P* = 0.023). While there were no significant difference in sex, follow-up time, BMI, average operative time, blood loss rate between the two groups (*P* > 0.05).

**Table 1:** Demographic and operative data, Group 1 versus Group 2

Patients’ characters	Group 1 (*n* = 19)	Group 2 (*n* = 15)	*P* value
Sex (boys/girls)	13/6	11/4	1.000
Age, month	42.26 ± 9.94 (26–58)	92.47 ± 20.37 (61–119)	0.000
BMI	15.82 ± 1.09 (13.89–18.08)	16.86 ± 3.95 (11.36–27.55)	0.277
follow-up time, month	45.11 ± 18.12 (24–80)	57.67 ± 24.03 (24–100)	0.092
The side of HB (right/left)	9/10	7/8	1.000
Operative time, min	163.42 ± 39.48 (75–242)	167.73 ± 34.58 (120–245)	0.741
Intraoperative blood loss, ml	260.00 ± 174.93 (50–600)	496.67 ± 393.91 (100–1600)	0.025
Blood loss rate, %	23.78 ± 17.59 (4.17–72.73)	26.35 ± 16.41 (6.06–53.33)	0.666
Fusion levels, segments	2.74 ± 1.05 (2–5)	3.60 ± 1.18 (2–5)	0.031
total main curve, °	48.58 ± 14.63 (21–80)	43.73 ± 10.59 (27–68)	0.289
segmental curve, °	44.16 ± 10.58 (21–68)	43.73 ± 10.59 (27–68)	0.908
Segmental kyphosis, °	27.50 ± 16.57 (8–56) 12	29.00 ± 11.57 (10–50) 13	0.794

### Radiographic deformity correction

**Fig. 1:**
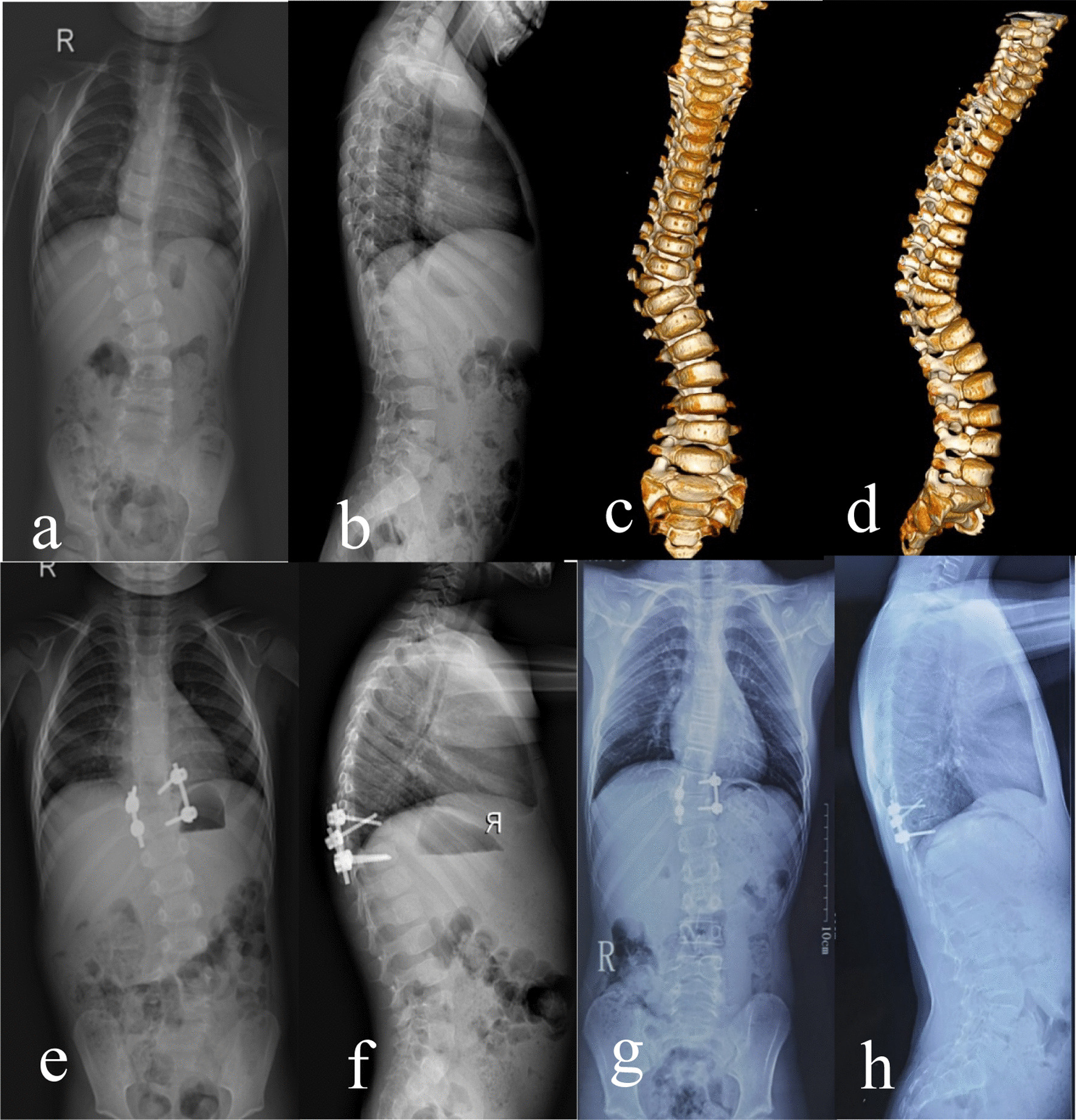
A 3 years 2 months boy with nonincarcerated thoracolumbar hemivertebra. Radiographs obtained preoperatively (**a**, **b**, X ray), (**c**, **d**, CT scan), postoperatively (**e**, **f**) and at the latest follow-up, 8 years later (**g**, **h**)

The radiographic correction details are summarized in Tables 2 and 3. Figure 1 shows one case from our study population.

**Table 2:** The radiological parameter change between pretreatment and IMPO (P1), and that between IMPO and latest follow-up (P2)

Radiographic characteristics	Pre-operation	IMPO	Last follow-up	p1	p2
Total main curve, °					
Group 1	48.58 ± 14.63 (21–80)	15.53 ± 7.57 (3–30)	19.165 ± 10.37 (2–42)	0.000	0.044
Group 2	43.73 ± 10.59 (27–68)	11.33 ± 9.59 (2–35)	12.13 ± 9.96 (0–28)	0.000	0.713
Segmental main curve, °					
Group 1	44.16 ± 10.58 (21–68)	11.53 ± 6.15 (3–22)	16.58 ± 9.97 (2–42)	0.000	0.010
Group 2	43.73 ± 10.59 (27–68)	11.33 ± 9.59 (2–35)	12.13 ± 9.96 (0–28)	0.000	0.713
Cranial compensatory curve, °					
Group 1 (*n* = 17)	16.29 ± 6.20 (9–33)	5.94 ± 5.29 (0–19)	7.41 ± 5.05 (0–22)	0.000	0.133
Group 2 (*n* = 13)	17.31 ± 5.56 (8–26)	6.54 ± 5.37 (0–19)	8.92 ± 6.26 (2–21)	0.000	0.077
Caudal compensatory curve, °					
Group 1 (*n* = 18)	26.78 ± 11.34 (13–55)	10.61 ± 9.51 (0–36)	9.78 ± 11.91 (-17–33)	0.000	0.629
Group 2 (*n* = 13)	20.85 ± 11.50 (5–39)	6.85 ± 6.62 (0–19)	6.31 ± 5.91 (0–17)	0.000	0.666
Segmental kyphosis, °					
Group 1 (*n* = 12)	27.50 ± 16.57 (8–56)	8.42 ± 6.50 (3–23)	9.50 ± 6.40 (0–19)	0.000	0.587
Group 2 (*n* = 13)	29.00 ± 11.57 (10–50)	5.00 ± 5.99 (-8–18)	8.00 ± 10.71 (-7–38)	0.000	0.292
Thoracic kyphosis, °					
Group 1	33.53 ± 12.30 (16–68)	28.79 ± 11.87 (9–56)	27.74 ± 11.30 (10–52)	0.194	0.747
Group 2	25.87 ± 16.47 (1–59)	26.33 ± 8.83 (16–50)	32.07 ± 15.54 (9–54)	0.899	0.082
Lumbar lordosis, °					
Group 1	52.16 ± 10.35 (34–74)	49.21 ± 6.29 (24–62)	52.37 ± 7.96 (35–66)	0.220	0.057
Group 2	51.87 ± 13.56 (28–75)	49.20 ± 14.06 (25–73)	52.87 ± 12.75 (33–82)	0.598	0.242
Absolute value of CB, mm					
Group 1	13.74 ± 9.05 (0–34)	10.63 ± 10.38 (0–34)	10.79 ± 7.15 (0–26)	0.356	0.951
Group 2	10.87 ± 5.58 (3–21)	10.53 ± 8.30 (0–25)	6.60 ± 6.49 (0–23)	0.889	0.039
Absolute value of SB, mm					
Group 1	26.58 ± 19.61 (3–69)	24.89 ± 14.04 (8–57)	25.16 ± 13.70 (2–58)	0.750	0.954
Group 2	29.73 ± 19.16 (2–65)	26.40 ± 18.03 (2–66)	22.33 ± 18.54 (0–63)	0.416	0.448

**Table 3:** Correction rate of IMPO and last follow-up according to the age at surgery

Patients’ characters	Group 1	Group 2	*P* value
Total main curve, °			
IMPO	68.05 ± 11.82 (51.35–92.86)	75.43 ± 16.67 (37.50–94.87)	0.140
Last follow-up	59.97 ± 20.23 (9.38–94.44)	73.94 ± 20.10 (34.88–100)	0.054
Segmental main curve, °			
IMPO	74.64 ± 11.04 (57.14–92.86)	75.43 ± 16.67 (37.50–94.87)	0.869
Last follow-u8p	62.89 ± 20.56 (9.38–94.44)	73.94 ± 20.10 (34.88–100)	0.126
Cranial compensatory curve, °(SD)			
IMPO	65.19 ± 25.59 (25–100)	64.69 ± 21.63 (20.83–100)	0.955
Last follow-up	53.72 ± 28.05 (6.25–100)	50.10 ± 32.23 (27.27–87.50)	0.745
Caudal compensatory curve, °(SD)			
IMPO	64.24 ± 24.34 (10.34–100)	64.42 ± 26.39 (21.05–100)	0.985
Last follow-up	57.31 ± 31.39 (-21.43–100)	71.36 ± 25.88 (31.58–100)	0.197
Segmental kyphosis, °(SD)			
IMPO	67.40 ± 13.03 (47.22–90)	84.73 ± 36.44 (10–180)	0.133
Last follow-up	60.85 ± 26.25 (20–100)	72..93 ± 44.58 (0–170)	0.423

In the coronal plane, the preoperative main curve of the group1 improved from 48.58° to 15.53° (68.05%) immediate postoperatively and 19.16° (59.97%) at last follow-up. In the group 2, the preoperative main curve improved from 43.73° to 11.33° (75.43%) and 12.13 (73.94%) at last follow-up. There was a loss of correction of 6.16% in group 1 and 1.49% in group 2 at last follow-up compared with the immediately postoperative values. The correction of the main curve immediately postoperative and at last follow-up in group 2 was better than that in group 1, but the difference was not significant. The correction rate of segmental scoliosis was 74.64%, from 44.16° pre-operation to 11.53° postoperation and 16.58° with a correction rate of 62.89% at last follow-up in group 1. In the group 2, The correction rate of segmental scoliosis was 75.43%, from 43.73° pre-operation to 11.33° postoperation and 12.13° with a correction rate of 73.94% at last follow-up. There was a loss of correction of 11.75% and 1.49% at last follow-up compared with the immediate postoperative values, respectively. Significant statistical differences were found in the coronal segmental and main curves between pre- and postoperative in both groups, and that between postoperative and at the last follow-up in group 1, but no significant statistical differences were found between postoperative and at the last follow-up in group 2. No significant statistical differences were found in the coronal segmental correction rate between the two groups.

In the group 1, the cranial compensatory correction rate was 65.19% immediate postoperatively and 53.72% at last follow-up. In the group 2, the correction rate was 64.69% and 50.10% respectively. There was a loss of correction of 11.47% in group 1 and 14.59% in group 2 during the follow-up period. The preoperative caudal compensatory curve improved 64.24% immediate postoperatively and 57.31% at last follow-up in the group 1. In the group 2, the correction rate was 64.42% immediate postoperatively and 71.36% at last follow-up. The improvement after surgery was significant (*P* = 0.000) in both groups, there was a loss of correction of 6.93% in group 1, and an improvement of 6.94% in group 2 during the follow-up, but no significant statistical differences were found. There was no significant difference of compensatory curve immediate postoperatively and at last follow-up (*P* > 0.05) between the two groups.

In the sagittal plane, some of the patients showed kyphotic deformity before surgery (12 in group1, 13 in group2). The segmental kyphosis of group 1 averaged 27.50°before surgery, 8.42° after surgery, and 9.50° at the last follow-up, with an average correction rate of 67.40% and 60.85%, respectively. The segmental kyphosis of group 2 averaged 29.00° before surgery, 5.00° after surgery, and 8.00° at the last follow-up, with an average correction rate of 84.73% and 72.93%, respectively. There was no significant difference of segmental kyphosis immediate postoperatively and at last follow-up (*P* > 0.05) between the two groups. In both groups, the average preoperative TK and LL were within normal range, and this was maintained immediate postoperatively and last follow-up. The average TK showed no significant changes during the follow-up period in both groups, but it slightly decreased in group 1, while it increased in group 2. The LL was maintained at last follow-up and showed no significant changes during the follow-up period in both groups, but it decreased slightly immediate postoperatively in both groups. The values of the LL were less influenced by the hemivertebra resection because of the compensation with the adjacent segments [11].

### Complications

There were no major vascular or neurological complications in this study. In all 34 patients, no deep wound infection, pseudarthrosis, implant failure was noted during the follow-up. One case in group 1 developed delayed wound union, debridement was performed, and the wound healed 3 weeks after surgery. There was a delayed superficial wound infection occurred 3 months after surgery in group 2, and debridement surgeries were needed. In group 1, four patients developed distal adding-on during the follow-up and 1 patients had proximal junctional kyphosis (PJK) postoperative and at the follow-up. In group 2, 3 patients were found proximal junctional kyphosis phenomenon, one of them with distal adding-on and a new curve in the cranial segments, all of them were under close observation and no additional surgery was needed until now.

## Discussion

The nonincarcerated hemivertebra has been recognized as a risk factor to develop a severe deformity with the growth of the spine. Early surgical treatment is widely suggested [8, 10, 15]. However, earlier hemivertebra resection with short segments fusion had the risk of less certain results in terms of correction and balance during the follow-up, as well as complications due to immaturity [7, 15, 16], such as pedicle fracture and displacement of screw, even damage of the spinal cord, nerve, vessel, wound complications because of the weak muscles and fascia of the infant. Furthermore, repeated or prolonged use of general anesthesia and sedatives in children under 3 years of age may affect the development of the child's brain [17]. But delayed treatment at a later stage may potentially require a long fusion involving more levels which could affect the spine function [18]. So, we believe that choosing a proper surgery technique and timing for certain patients individually is very important.

Chang et al. [19] Compared the Surgical outcomes by age at the time of surgery in the treatment of congenital scoliosis in children under age 10 years, they concluded that having surgery before 6 years old had significantly better deformity correction and did not cause a negative effect on the growth of vertebral body or spinal canal compared with the group treated after 6 years of age. While in their study, the type and location of hemivertebra were not unified, which will affect the progression of the deformity. In our study, all the patients were diagnosed as scoliosis due to single nonincarcerated thoracolumbar hemivertebra, we found that the preoperative coronal main curve, segmental curve and caudal compensatory curve in the group 1 were slightly larger than that in the group 2, and the cranial compensatory curve and segmental kyphosis were larger in the group 2 than in the group 1, but the difference between the two groups were not statistically. This suggested that not all the deformities caused by nonincarcerated thoracolumbar hemivertebra would progress greatly with the spinal growth, especially when the main curve was the segmental curve, such as cases of group 2 in our series.

Sanchez-Marquez et al. [9] reported early thoracolumbar and lumbosacral hemivertebra resection and transpedicular short fusion in patients younger than 5 years allowed for good coronal correction immediately postoperative with a correction rate of 68%, which was difficult to maintain with a correction rate of 56% at midterm follow-up. According to the results of our study, There was a better correction of main curve, segmental curve, caudal compensatory curve and segmental kyphosis in the group 2 immediate postoperatively and at last follow-up compared with that of the group 1. This implies that group 2 may have more acceptable radiologic outcomes for deformity correction in our series. This result is different with the previous study reported by Chang et al. [19] above that was possibly because in our study the fusion segments in the group 2 were more than that in the group 1 (3.60 and 2.74, respectively), in this sense, we hold that it is worth to have a little longer fusion in a delayed surgery to get a better correction.

There were no major vascular or neurological complications in our study. The main problem was the loss of correction, emerging adding-on and PJK. Chang et al. [20] reported that the cause of revision surgery for the curve progression may include inappropriate fusion level, incomplete hemivertebra resection, or failure of fusion. In our series, 6 cases developed distal adding-on in the last follow-up, 5 of them in the group 1 had bisegmental fusion, and 1 of them in the group 2 together with a new emerging cure in the cranial segment had a fusion of five segments. We found that among the 6 cases with adding-on, 5 of them had the hemivertebra located in thoracolumbar junction (3 with T12 HV and 2 with T12/L1 HV), we speculate that younger age less than 5 years, short segment fusion and hemivertebra located in thoracolumbar junction might be the risk factors of adding-on after hemivertebra resection in younger children of congenital scoliosis.

PJK after posterior hemivertebra resection and short fusion in young children with congenital scoliosis was well studied by Chen et al. [21], they concluded that the incidence of PJK caused by thoracolumbar hemivertebra was 10.3%, and the risk factors including preoperative segmental kyphosis, number of fusion levels, a larger postoperative SVA and ligamentous failure. The incidence of PJK in our study was 8.82%, they all have the risks of 3 to 5 fusion levels and preoperative segmental kyphosis near or more than 30 degree, which were consistent with previous reports. Concluding the risk factors above, we found that the fusion level was a contrary factor for adding-on and PJK, so we recommend that the preoperative segmental kyphosis should be taken into consideration when choosing the fusion levels. We agree with the strategies to avoid PJK given by previous reports, including protection of the soft tissue, proper shaped rods, and brace treatment at least 6 months after surgery [21, 22].

This study has several limitations. First, most of the cases in present study had not yet reached bone maturity at the last follow-up. Second, this is a retrospectively reviewed study with small number of patients included, continuous follow-up and more patients are needed in the future. The third limitation is that the patients were too young to fill out questionnaires of Health-related quality of life by themselves.

## Conclusion

Not all the deformities caused by single nonincarcerated thoracolumbar hemivertebra would progress greatly with the spinal growth, especially when the main curve was the segmental curve. Posterior hemivertebra resection with transpedicular instrumentation is a safe and effective procedure in treatment of this kind of congenital scoliosis. No significant statistical differences were found in the coronal and sagittal correction rate between the two groups. A limited delayed surgery after 5 years but before 10 years of age with close follow-up can achieve satisfied correction and lessen the problem of unable to cooperate due to age, meanwhile. As most of the patients in our study are still young, continuous follow-up is needed in the further.

## Data Availability

The datasets used and/or analyzed during the current study are available from the corresponding author on reasonable request.

## Abbreviations

HV: Hemivertebra
AP: Anterior–posterior
SK: Segmental kyphosis
TK: Thoracic kyphosis
LL: Lumbar lordosis
EBV: Estimated blood volume
SEP: Somatosensory evoked potential
MEP: Motor-evoked potential
PJK: Proximal junctional kyphosis
SVA: Sagittal vertebral axis
CB: Coronal balance
SB: Sagittal balance
IMPO: Immediately postoperative
